# Motor competence explains the variance in biomechanical variables related to anterior cruciate ligament injury risk, with distinct predictors for male and female athletes

**DOI:** 10.1002/jeo2.70531

**Published:** 2026-01-14

**Authors:** Behzad Mohammadi Orangi, Aref Basereh, Mansoureh Shahraki, Altay Ulusoy, Paul A. Jones

**Affiliations:** ^1^ Department of Sport Science, School of Humanities Damghan University Damghan Iran; ^2^ Faculty of Physical Education and Sport Sciences Kharazmi University of Tehran Tehran Iran; ^3^ Department of Sport Sciences, Faculty of Literature and Humanities University of Zabol Zabol Iran; ^4^ Department of Exercise and Sport Sciences, School of Physical Education and Sports Istanbul Nişantaşı University Istanbul Turkey; ^5^ Centre for Health Sciences Research, School of Health & Society University of Salford Manchester UK

**Keywords:** athlete, biomechanics, kinematic variables, kinetic variables, motor skill

## Abstract

**Purpose:**

Anterior cruciate ligament (ACL) injuries have consistently been linked to specific kinetic and kinematic patterns, including elevated vertical ground reaction forces, increased knee abduction angle and moment (dynamic valgus), reduced knee flexion during landing and excessive hip adduction/internal rotation. However, the relationship between motor competence as a factor affecting athletes' performance and kinetic and kinematic variables has not yet been investigated.

**Methods:**

A total of 112 elite athletes (66 males and 46 females; mean age = 19.4 ± 1.1 years) from basketball, volleyball and handball were assessed. Motor competence was evaluated using the short form of the Bruininks–Oseretsky Test of Motor Proficiency. Biomechanical data were collected during a single‐leg drop landing task using a three‐dimensional motion analysis system (Vicon) and a force plate. Pearson correlation coefficients and multiple linear regression (Enter method) were used to analyse relationships between motor competence and kinetic/kinematic variables.

**Results:**

All biomechanical variables showed significant correlations with motor competence (*p* < 0.001). Notably, knee flexion angle (*r* = 0.613) and knee abduction angle (*r* = −0.576) demonstrated strong associations. Regression analysis identified several biomechanical variables that were statistically associated with motor competence, explaining 69.6% of its variance.

**Conclusion:**

Motor competence was related to several kinetic and kinematic variables previously linked to ACL injury risk. However, due to the cross‐sectional design, these associations should not be interpreted as causal, and further longitudinal or interventional studies are warranted.

**Level of Evidence:**

Level IV, cross‐sectional study.

**Abbreviations**
ACL: anterior cruciate ligament
BMI: body mass index
BOT‐2 SF: Bruininks–Oseretsky Test of Motor Proficiency
PADA: peak ankle dorsiflexion
PHAA: hip adduction angle
PHAM: hip abduction moment
PHERM: hip external rotation moment
PHFA: hip flexion angle
PHIRA: hip internal rotation angle
PKAA: knee abduction angle
PKAM: knee abduction moment
PKFA: knee flexion angle
SL‐DVJ: single‐leg vertical drop jump task
vGRF: peak vertical ground reaction force

## INTRODUCTION

Several factors have been suggested to contribute to the risk of anterior cruciate ligament (ACL) injury [33, 35]. Among these factors are kinetic and kinematic variables during athletic tasks, which play a key role in modulating ACL injury risk [2, 18, 19, 28]. Kinetic variables describe the forces and moments acting on the lower limb. A lower peak vertical ground reaction force (vGRF) during landing indicates better muscular force absorption and reduced stress on the knee. Greater hip abduction moment enhances pelvic stability and reduces lateral knee loading, while increased hip external rotation moment improves hip stability and control of rotational forces. Conversely, higher knee abduction angle and moment are associated with greater lateral knee forces and are recognised as major contributors to non‐contact ACL injuries [2, 3, 7, 18, 19]. Kinematic variables reflect joint movement angles and their changes during motion [2]. A larger ankle dorsiflexion angle and knee flexion angle allow for more effective impact absorption and lower stress on knee structures. Similarly, increased hip flexion angle improves shock absorption and stability, whereas reduced hip adduction and internal rotation angles decrease the likelihood of unsafe knee positioning and harmful loading [2, 3, 18, 19]. So by first identifying factors related to these variables and then suggesting possible interventions to strengthen them, it may be possible to mitigate ACL injuries to some extent.

Given the key role of kinetic and kinematic variables in ACL injury, examining the factors that influence these variables is of particular importance. One such factor is motor competence (MC), defined as the ability to skillfully and coordinately perform fundamental movement skills, maintain postural control, and adapt to environmental demands [6, 8]. This concept includes abilities such as balance, coordination, and control of complex movements, all of which are essential in sports and daily life [6, 27]. Recent evidence also confirms that balance, a core component of motor competence, can be reliably assessed during demanding tasks such as single‐limb stance, further highlighting its role in postural control [33]. Stodden et al. proposed that individuals with higher motor competence demonstrate greater control over their movements, leading to improved athletic performance and better adaptability in dynamic contexts [31]. In line with this, Dynamic Systems Theory posits that movement emerges from the interaction of the body's biomechanical–neurological systems, the environment, and the task; within this framework, individuals with higher motor competence provide more optimal motor responses [5]. Furthermore, Neuromuscular Control Theory highlights that more precise muscle coordination during complex actions results in more efficient movement patterns and a more favourable distribution of forces [20, 26, 30]. These characteristics may contribute to reducing vertical forces, better controlling lateral forces, and improving joint angle regulation, kinetic and kinematic variables directly associated with ACL injury. Nevertheless, the relationship between motor competence and these variables still requires further empirical investigation. Therefore, this study aimed to examine whether motor competence is associated with biomechanical risk factors for ACL injury in athletes of basketball, volleyball, and handball. It was hypothesised that higher motor competence would be linked to a more favourable biomechanical profile.

## MATERIALS AND METHODS

This study was designed as a cross‐sectional investigation and was approved by the Ethics Committee of the University of Zabol (IR.UOZ.REC.1403.036). All participants provided written informed consent prior to enrolment, and patient selection was performed according to the following inclusion and exclusion criteria.

### Participants

Participants were required to meet the following inclusion criteria: (1) Male and female handball, volleyball, and basketball players. (2) Age between 18 and 21 years. (3) A normal body mass index (BMI) within the range of 18.5–25. (4) No history of fractures or dislocations in the hip, knee, or ankle. (5) No history of lower back pain within the past year (to avoid confounding effects on posture and lower‐limb biomechanics during landing tasks). (6) No vestibular or ligament injuries in the lower limb [13, 29]. (7) Having a motor competence score above the fifth percentile (below this percentile is suspected of developmental coordination disorder). Participants were excluded if they met any of the following criteria: (1) Voluntary withdrawal from the study. (2) Failure to meet the inclusion criteria at any point during the study. (3) Lower limb deformities such as genu varum (knee spacing greater than 3 cm between the condyles), genu valgum (ankle spacing greater than 3 cm), or genu recurvatum (hyperextension of the knee) [13, 29].

Based on the Participant Classification Framework proposed by McKay et al. [23], the participants in this study were classified as Tier 2: Trained/Developmental athletes. These athletes regularly competed at university, provincial, or local levels and engaged in structured training sessions approximately three times per week with the purpose of improving performance and progressing toward higher competitive levels. Consistent with the Tier 2 category, they were involved in organized teams such as sports academies, university l leagues, and provincial programs, and were actively developing the skills required to advance to national‐level competition.

## OUTCOME MEASURES

### Single‐leg vertical drop jump task (SL‐DVJ)

**Figure 1:**
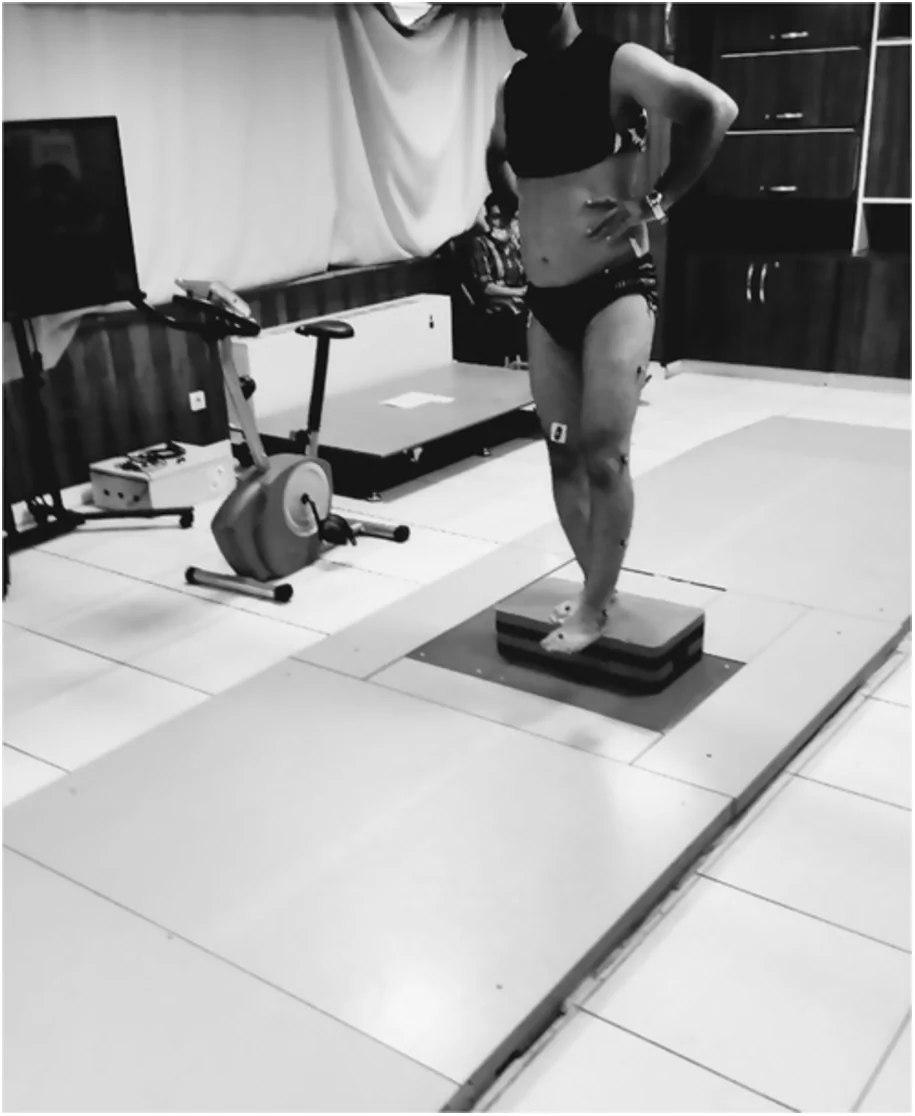
Single‐leg vertical drop jump task.

To determine the dominant foot, participants were asked which foot they preferred to land on after a jump. According to Figure 1, the SL‐DVJ task involved landing from 30 cm high box, landing on the dominant foot, immediately performing a maximal vertical jump, and landing again [14, 22]. If participants jumped off the box instead of landing from it, if the opposite foot touched the ground, if they clearly lost balance, or if they fell during the test, the trial was discarded and repeated. Additionally, a one‐minute rest was provided between each trial to prevent fatigue.

Three‐dimensional kinematic and kinetic data were synchronously collected using eight cameras from the Vicon Motion System (sampling frequency: 120 Hz) and a force plate (Watertown, MA) installed flush with the laboratory floor (sampling frequency: 1200 Hz). After performing the static calibration test, markers were placed according to previous studies on specific anatomical landmarks (left and right front and back of head (on a headband), 7th cervical vertebrae, 10th thoracic vertebrae, clavicle, sternum, right back, shoulders, lateral epicondyles of elbow, medial wrists, lateral wrists, second metacarpal heads, anterior superior iliac spines, posterior superior iliac spines, lateral thighs, lateral epicondyles of knee, lateral shanks, lateral malleoli, second metatarsal heads, and heels) [32]. The Vicon Plug‐in‐Gait model was used to obtain lower extremity kinematic data. A right‐handed global laboratory coordinate system was defined during Vicon static calibration. Axes were oriented as +X anterior (forward), +Y to the participant's left (mediolateral), and +Z vertical (upward). Ground‐reaction forces and moments from the force plate were aligned to, and exported in, this global frame by Vicon Nexus. Segment coordinate systems were generated by the Plug‐in‐Gait model from anatomical landmarks (pelvis: ASIS/PSIS; thigh: hip–knee; shank: knee–ankle; foot: heel–toe markers). Joint kinematics were expressed using an ISB‐recommended Cardan sequence X–Y–Z (flexion/extension, ab/adduction, internal/external rotation). External joint moments (Nm/kg) were obtained via Plug‐in‐Gait inverse dynamics and reported in the global frame. For clarity, in the results knee abduction angle (valgus) and knee abduction moment (external) are referred to as positive in the frontal plane; vertical GRF is reported as positive (upward). To reduce noise, all kinematic and force plate data were filtered using a fourth‐order low‐pass Butterworth digital filter with a cutoff frequency of 12 Hz. The choice of cut‐off frequency was based on visual inspection of several cut‐off frequencies. The maximum ground reaction forces in the vertical and posterior directions, hip abduction and external rotation moments, and maximum flexion angles of the hip, knee, ankle dorsiflexion, and knee abduction during landing were calculated. The average of three successful attempts was recorded as the participants' score. MATLAB engineering software (Version 8.4, 2014b) was used for data analysis.

### BOT‐2 test

To evaluate MC, the short form of the Bruininks‐Oseretsky Test of Motor Proficiency (BOTMP‐2SF) [4], developed for 4–21 year old participants, was used. The BOTMP‐2 is a standardised and individually administered test battery to measure MC, consisting of 53 items in the full version or 14 items in the short form. Items are organised into four motor area composites: (a) fine manual control (fine motor precision and fine motor integration e.g. drawing lines through paths), (b) manual coordination (manual dexterity and upper‐limb coordination, e.g. dribbling a ball), (c) body coordination (bilateral coordination and balance, e.g. standing heel‐toe on a beam), and (d) strength and agility (running speed, agility, and strength, e.g. hopping on one leg). Its maximum Total Point Score (or raw score) is 88, and its range of standard score is 20–80. The participants were evaluated strictly according to the guidelines of the BOTMP‐2SF manual [9]. The point scores of the four motor‐area composite scores were combined in a total motor composite score and converted into an age and sex standardised MC score. The test has demonstrated validity and reliability, with a reliability coefficient of 90% for motor skills assessment. The retest reliability coefficients are 0.78 for the long form and 0.86 for the short form. The short form assesses overall motor skills, with the total score reflecting a combination of gross and fine skills [4, 9]. The BOT‐2 SF is considered one of the most widely used and valid standardised assessments of motor competence, covering a broad age range (4–21 years). Recent studies have also applied the BOT‐2 in late adolescents and young adults within this age group, supporting its suitability for the present sample [16, 24].

The test was completed indoors in gym facilities. Hand and foot preference were determined by means of a ball throwing and kicking task according to the BOTMP‐2SF manual. After describing the purpose of each test, supplemented by additional verbal information or a demonstration, if necessary, each participant was allowed one test trial for each test item. In addition, each test was video recorded from the sagittal and frontal planes. After the test, a total score for each participant was obtained and reported. Two evaluators independently scored each participant in real life [15, 16, 17, 24], and from the two data series, interrater reliability was calculated and found very high (87.4% agreement). However, if there was a difference in the scores of the first and second evaluator, the score of the first evaluator was used for further analysis due to the higher experience and expertise of the first researcher. A third evaluator calculated the point scores from the raw scores for each item, as well as the overall point score (range: 0–88 points). The second author converted the total point scores into standardised scores (range 20–80) according to the manual guidelines. According to the Bruininks‐Oseretsky test, a standard score below the fifth percentile rank (20–33) is indicative of developmental coordination disorder. Therefore, in the present study, all participants were required to have a score above the fifth percentile to be included.

### Statistical analysis

Based on calculations performed using G*Power software, and considering an effect size of 0.15, an alpha error probability of 0.05, and a test power of 0.95, the required sample size for this study was determined to be 89 participants. The effect size was selected based on a review of existing literature [12, 16]. To account for an estimated dropout rate of approximately 20%, a total of 112 athletes (66 males and 46 females) from basketball, volleyball, and handball from the Iran were recruited for participation. Statistical analyses were conducted after confirming that the data were normally distributed using the Shapiro–Wilk test. The differences in the characteristics between the males and females were determined using an independent t‐test. Correlations between motor competence and biomechanical variables were assessed using Pearson's correlation coefficient. A standard multiple regression analysis was conducted to predict motor competence from biomechanical variables. All statistical analyses were performed using SPSS version 24. Differences and correlations were considered significant when *p* < 0.05. To enhance the interpretability of the correlation results, the strength of the associations was categorised based on the guidelines by Mukaka [25]. According to this classification, correlation coefficients were interpreted as follows: values between 0.00–0.10 (negligible), 0.10–0.29 (weak), 0.30–0.49 (moderate), and ≥0.50 (strong).

## RESULTS

Initially, 121 athletes were recruited, but nine athletes were subsequently excluded (due to prior knee injury [three athletes] and not being within the age range of the study [six athletes]) prior to data collection, leaving 112 athletes. The mean ± SD age, mass and height of the participants was; 19.4 ± 1.1 years, 68.3 ± 6.5 kg 178.2 ± 7.4 cm, respectively.

### Gender differences in biomechanical variables and motor competence

To examine gender differences across the studied variables, an independent samples t‐test was conducted. Prior to this analysis, the normality of data distribution for each variable within the male and female groups was confirmed using the Shapiro–Wilk test (*p* > 0.05). The results indicated no statistically significant differences (*p* > 0.05) between males and females in any of the variables (Table 1).

**Table 1:** Independent t‐test results for gender comparison across study variables.

	*t*	*df*	*p* value	Cohen's *d*	Test of equality of variances (Levene's)
vGRF (N/kg)	−0.696	110	0.488	−0.134	0.331
PADA (**°**)	0.018	110	0.986	0.003	0.391
PHAM (N/kg)	−0.967	110	0.336	−0.186	0.617
PHAA (**°**)	−0.907	110	0.366	−0.174	0.837
PHERM (N/kg)	−0.527	110	0.599	−0.101	0.294
PHFA (**°**)	−0.008	110	0.994	−0.002	0.914
PHIRA (**°**)	1.820	110	0.072	0.349	0.889
PKAA (**°**)	0.198	110	0.843	0.038	0.979
PKAM (N/kg)	0.439	110	0.661	0.084	0.337
PKFA (**°**)	1.440	110	0.153	0.277	0.211
MC (score)	1.151	110	0.252	0.221	0.915

Abbreviations: MC, motor competence; PADA, peak ankle dorsiflexion; PHAA, hip adduction angle; PHAM, hip abduction moment; PHERM, hip external rotation moment; PHFA, hip flexion angle; PHIRA, hip internal rotation angle; PKAA, knee abduction angle; PKAM, knee abduction moment; PKFA, knee flexion angle; vGRF, peak vertical ground reaction force.

### Correlations between motor competences

**Figure 2:**
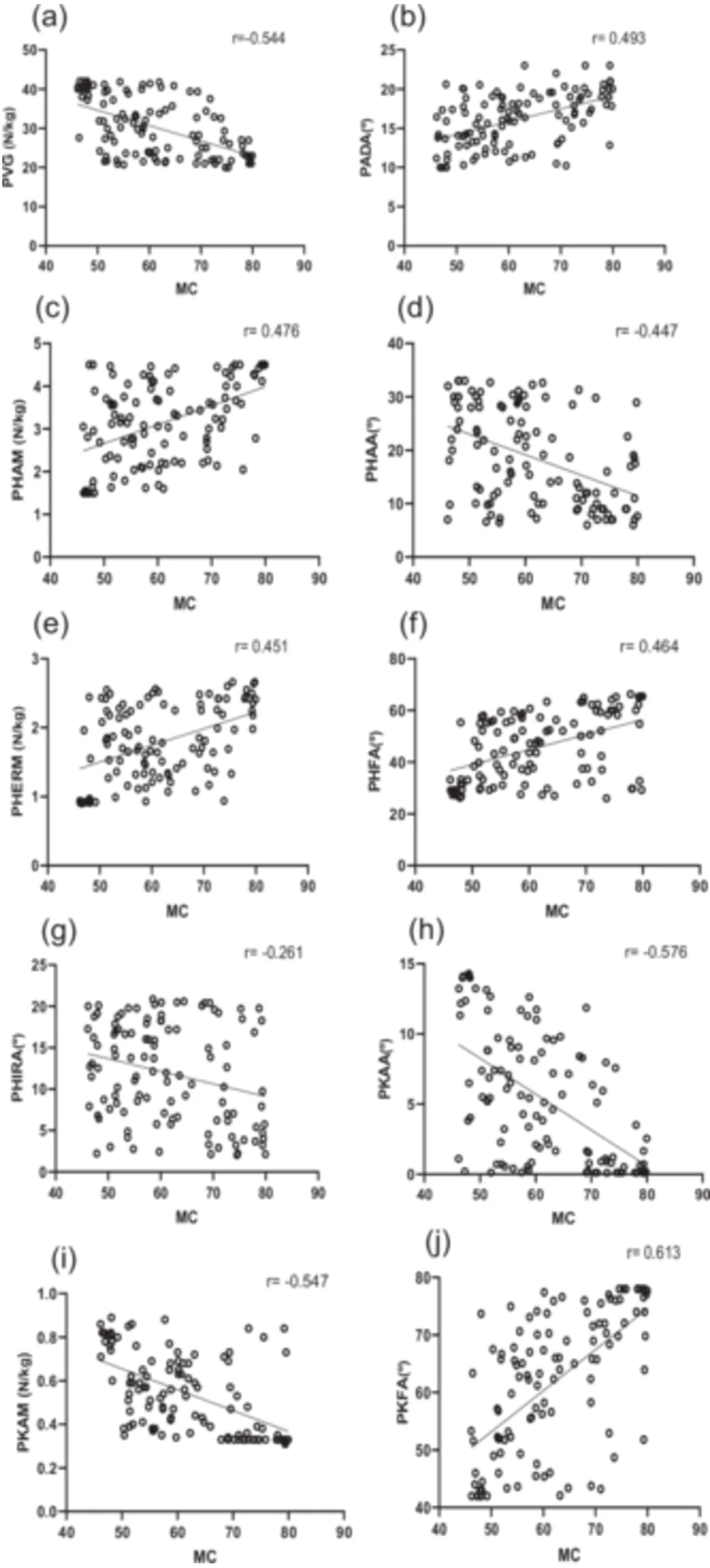
Relationships between motor competence and (a) peak vertical ground reaction force (vGRF), (b) peak ankle dorsiflexion (PADA), (c) hip abduction moment (PHAM), (d) hip adduction angle (PHAA), (e) hip external rotation moment (PHERM), (f) hip flexion angle (PHFA), (g) hip internal rotation angle (PHIRA), (h) knee abduction angle (PKAA), (i) knee abduction moment (PKAM) and (j) knee flexion angle (PKFA).

Pearson correlation coefficients were calculated to assess the relationships between motor competence and biomechanical variables. All variables showed statistically significant correlations with motor competence (*p* < 0.001). The highest correlation was observed for peak knee flexion angle (PKFA) with a strong association (*r* = 0.613), followed by peak knee abduction angle (PKAA) and peak knee abduction moment (PKAM), both also showing strong associations (*r* = −0.576 and *r* = 0.547, respectively). VGRF and peak ankle dorsiflexion angle (PADA) showed strong and moderate correlations, respectively (*r* = 0.544 and *r* = 0.493). Peak hip flexion angle (PHFA), peak hip external rotation moment (PHERM), and hip adduction angle (PHAA) each demonstrated moderate associations. The lowest correlation was found with hip internal rotation angle (PHIRA), which indicated a weak correlation (*r* = 0.261) (Figure 2).

### Multiple regression analysis predicting motor competence

The multiple regression analysis revealed that the biomechanical variables model significantly predicted approximately 70% of the variance in motor competence (*R*² = 0.696, *F* = 28.931, *p* < 0.001). Among the ten predictors, seven variables showed significant relationships with motor competence: vGRF (*β* = −0.191, *p* = 0.004), PADA (*β* = 0.207, *p* = 0.002), PHAA (*β* = −0.175, *p* = 0.005), PHERM (*β* = 0.139, *p* = 0.030), PKAA (*β* = −0.150, *p* = 0.035), PKAM (*β* = −0.174, *p* = 0.011) and PKFA (*β* = 0.156, *p* = 0.033). The remaining three variables—PHAM, PHFA, and PHIRA—did not demonstrate significant effects (*p* > 0.05).

The multiple regression analysis for males revealed that the biomechanical variables model significantly predicted 70% of the variance in motor competence (*R*² = 0.700, *F* = 12.85, *p* < 0.001). Among the ten predictors, four variables showed significant relationships with motor competence: vGRF (*β* = −0.248, *p* = 0.006), PHAA (*β* = −0.188, *p* = 0.025), PHERM (*β* = 0.186, *p* = 0.045) and PKAM (*β* = −0.158, *p* = 0.018). The remaining variables PADA, PHAM, PHFA, PHIRA, PKAA and PKFA did not demonstrate significant effects (*p* > 0.05).

The multiple regression analysis for females revealed that the biomechanical variables model significantly predicted 76% of the variance in motor competence (*R*² = 0.760, *F* = 11.08, *p* < 0.001). Among the 10 predictors, two variables showed significant relationships with motor competence: PADA (*β* = 0.285, *p* = 0.004) and PKFA (*β* = 0.269, *p* = 0.023). The remaining variables did not demonstrate significant effects (*p* > 0.05).

## DISCUSSION

The purpose of the present study was to examine the relationship between motor competence and biomechanical variables including kinetic and kinematic variables associated with ACL injury risk in athletes participating in sports involving a high number of landing actions. The results showed, motor competence is closely intertwined with lower‐limb biomechanics. Also, nearly 70% of the variance in motor competence was explained by joint mechanics and ground reaction force variables. While no mean‐level sex differences were observed, the predictors differed: males relied more on kinetic control, whereas females showed stronger links with kinematic parameters.

The correlation analysis showed that all kinetic and kinematic variables were significantly associated with motor competence. Strong associations with motor competence were observed for knee flexion angle and knee abduction angle. Higher motor competence was correlated with more favourable biomechanical profiles such as improved force attenuation, more optimal joint angles, and reduced harmful lateral forces. Furthermore, the multiple regression analysis indicated that approximately 70% of the variance in motor competence could be predicted by vertical ground reaction force, ankle dorsiflexion angle, hip adduction angle, hip external rotation moment, knee abduction angle, knee abduction moment and knee flexion angle. Collectively, these findings indicate an association between motor competence and the quality of movement execution. It is possible that individuals with higher motor competence employ more stable, coordinated, and efficient motor strategies during dynamic tasks, though this requires confirmation in longitudinal studies. The underlying reasons may include greater neuromuscular coordination, richer motor experience, and more refined control over complex movements [6, 10]. Additionally, such individuals may naturally adopt safer and more efficient movement patterns when responding to mechanical demands. It should also be noted that hip internal rotation angle showed only a weak correlation with motor competence, suggesting that this variable may be less sensitive to differences in motor competence and more influenced by relatively stable structural or anatomical characteristics.

No significant differences were observed between male and female participants across the studied variables. This analysis was conducted solely to determine whether data from both sexes could be combined, and given the absence of significant differences, datasets were merged for subsequent analyses. Considering that all participants were high‐level, physically active athletes, this lack of difference was somewhat expected. Similar levels of training experience, structured athletic programmes and long‐term neuromuscular adaptations likely contributed to convergence in motor patterns and biomechanical indicators between males and females.

The sex‐stratified regression analyses revealed that although no mean‐level differences emerged between males and females, the predictors of motor competence were not the same. In males, vertical ground reaction force, hip adduction angle, hip external rotation moment, and knee abduction moment, played a more prominent role. By contrast, in females, ankle dorsiflexion (PADA) and knee flexion angle, were the key predictors. In the combined model, a mix of these variables reached significance, indicating that both kinetic regulation at the hip and knee (highlighted in males) and sagittal‐plane kinematics at the ankle and knee (highlighted in females) jointly contribute to motor competence. This suggests that while males tend to organise their motor competence through controlling forces and moments, females more often rely on movement excursions to absorb impact. Such differences may reflect anthropometric factors, neuromuscular activation strategies, and training backgrounds. Importantly, the absence of mean sex differences does not preclude sex‐specific weighting of predictors. These findings open a valuable avenue for future studies to further explore sex‐based mechanisms and to design tailored interventions that address these distinct movement strategies.

Overall, the findings of this study suggest that motor competence is associated with biomechanical indicators linked to ACL injury mitigation. Individuals with higher motor competence are better able to pre‐position their joints before ground contact and absorb impact forces through more efficient movement strategies involving the knee, ankle and surrounding musculature [6, 10]. These patterns are not merely a product of muscular strength, but rather reflect the quality of motor organisation [26]. This interpretation aligns closely with Stodden et al. [31] developmental model, which emphasises that higher motor competence supports more adaptive and efficient movement execution by enhancing neuromuscular coordination and control. According to this framework, individuals with greater motor competence demonstrate not only better movement outcomes but also improved readiness of the neuromuscular system to respond to dynamic task demands [6, 31].

From the lens of Ecological Dynamics theory [1, 11, 21, 34], motor competence is not viewed as a fixed internal trait, but as the emergent result of continuous interaction between the individual, the task, and the environment. Within this perspective, effective movement arises from one's ability to perceive and act in a way that adapts to contextual challenges. The results of this study, particularly those showing greater hip external rotation moment and reduced knee abduction moment in highly competent movers, support the idea that these individuals exhibit superior adaptability and control in destabilising scenarios. These characteristics reflect greater perceptual‐motor attunement and action flexibility—central tenets of the ecological approach. Thus, motor competence functions not only as an indicator of movement quality but also as a fundamental modulator of biomechanical risk factors associated with ACL injury under dynamic conditions.

## STRENGTHS AND LIMITATIONS

Despite the notable strengths and innovations of this study, certain limitations remain, although several strategies were implemented to minimise their impact. Given the cross‐sectional design, causal relationships cannot be firmly established. To partially address this, strict inclusion and exclusion criteria were applied, and participants were drawn from a homogenous athletic population, thereby enhancing internal validity. Although factors such as sex and skill level were controlled, other potential confounders including sport type, lower‐limb strength, landing experience, prior injury, and body composition, could not be fully accounted for. Efforts were made to mitigate this by selecting athletes with comparable training histories and by applying BMI restrictions, yet subtle differences may still have influenced results. The study utilised a single motor task (the single‐leg vertical drop jump). While this may limit generalisability, the task was deliberately chosen due to its wide acceptance as a biomechanically demanding and valid screening tool for ACL injury risk. Although psychological and neurological factors such as movement anxiety or central motor control were not directly measured, the inclusion of validated biomechanical variables such as joint angles and joint moments at the hip, knee, and ankle, provided robust indicators of movement quality. A standard multiple regression approach was employed, which limited the exploration of interactions and nonlinear patterns; however, this method allowed for a clear initial assessment of the collective predictive value of biomechanical variables. Although two independent assessors scored the BOT‐2, disagreements were resolved by the more experienced evaluator without a formal consensus procedure. This approach minimised inconsistency but may still introduce bias, warranting refinement in future studies. Finally, although not all mechanisms of ACL injury were captured, the multivariate approach ensured that a set of high‐impact, validated variables was analysed collectively, offering a comprehensive starting point for understanding the interplay between motor competence and injury‐related biomechanics. Future research should build on these efforts by incorporating additional confounders, multiple motor tasks, and advanced statistical models to further expand these insights.

## CONCLUSION

In this cross‐sectional study, motor competence was significantly associated with several biomechanical variables, with nearly 70% of its variance explained by joint mechanics and ground reaction forces. Although no mean sex differences were found, the predictors differed between males and females, suggesting distinct biomechanical pathways. These results indicate that motor competence may serve as a relevant marker of movement quality, and, with further longitudinal confirmation, could potentially help identify athletes at higher risk of ACL injury.

## AUTHOR CONTRIBUTIONS

*Conceptualisation*: Behzad Mohammadi Orangi. *Methodology*: Behzad Mohammadi Orangi and Mansoureh Shahraki. *Data collection*: Mansoureh Shahraki. *Data analysis*: Aref Basereh. *Writing—original draft preparation*: Behzad Mohammadi Orangi and Aref Basereh. *Writing—review and editing*: Behzad Mohammadi Orangi, Mansoureh Shahraki, Aref Basereh, Altay Ulusoy and Paul A. Jones. All authors have read and agreed to the published version of the manuscript. All authors have reviewed and approved the final version of the manuscript, and have consented to the order of authorship.

## CONFLICT OF INTEREST STATEMENT

The authors declare no conflicts of interest.

## ETHICS STATEMENT

This study was approved by the Ethics Committee of the University of Zabol (IR.UOZ.REC.1403.036). Participants provided written informed consent after the study's objectives were explained to them.

## Data Availability

Data are available to qualified investigators on reasonable request.
